# *Lactobacillus plantarum* PS128 Improves Physiological Adaptation and Performance in Triathletes through Gut Microbiota Modulation

**DOI:** 10.3390/nu12082315

**Published:** 2020-08-01

**Authors:** Wen-Ching Huang, Chun-Hsu Pan, Chen-Chan Wei, Hui-Yu Huang

**Affiliations:** 1 Department of Exercise and Health Science, National Taipei University of Nursing and Health Sciences, Taipei 11219, Taiwan; wenching@ntunhs.edu.tw; 2 Ph.D. Program in Biotechnology Research and Development, College of Pharmacy, Taipei Medical University, Taipei 11031, Taiwan; panch@tmu.edu.tw; 3 School of Pharmacy, Taipei Medical University, Taipei 11031, Taiwan; 4 Department of Aquatic Sport, University of Taipei, Taipei 11153, Taiwan; tom911072@gmail.com; 5 Graduate Institute of Metabolism and Obesity Sciences, Taipei Medical University, Taipei 11031, Taiwan

**Keywords:** *L. plantarum* PS128, gut microbiota, SCFAs, exercise, triathlete

## Abstract

A triathlon is an extremely high-intensity exercise and a challenge for physiological adaptation. A triathlete’s microbiome might be modulated by diet, age, medical treatments, lifestyle, and exercise, thereby maintaining aerobiosis and optimum health and performance. Probiotics, prebiotics, and synbiotics have been reported to have health-promoting activities (e.g., immunoregulation and cancer prevention). However, few studies have addressed how probiotics affect the microbiota of athletes and how this translates into functional activities. In our previous study, we found that *Lactobacillus plantarum* PS128 could ameliorate inflammation and oxidative stress, with improved exercise performance. Thus, here we investigate how the microbiota of triathletes are altered by *L. plantarum* PS128 supplementation, not only for exercise performance but also for possible physiological adaptation. The triathletes were assigned to two groups: an *L. plantarum* 128 supplement group (LG, 3 × 10^10^ colony-forming units (CFU)/day) and a placebo group (PG). Both groups continued with their regular exercise training for the next 4 weeks. The endurance performance, body composition, biochemistries, blood cells, microbiota, and associated metabolites were further investigated. PS128 significantly increased the athletes’ endurance, by about 130% as compared to the PG group, but there was no significant difference in maximal oxygen consumption (VO_2_max) and composition between groups. The PS128 supplementation (LG) modulated the athlete’s microbiota with both significant decreases (*Anaerotruncus*, *Caproiciproducens*, *Coprobacillus*, *Desulfovibrio*, *Dielma*, *Family_XIII*, *Holdemania*, and *Oxalobacter*) and increases (*Akkermansia*, *Bifidobacterium*, *Butyricimonas*, and *Lactobacillus*), and the LG showed lower diversity when compared to the PG. Also, the short-chain fatty acids (SCFAs; acetate, propionate, and butyrate) of the LG were significantly higher than the PG, which might be a result of a modulation of the associated microbiota. In conclusion, PS128 supplementation was associated with an improvement on endurance running performance through microbiota modulation and related metabolites, but not in maximal oxygen uptake.

## 1. Introduction

The triathlon competition originated in 1974 in San Diego, and includes three sports: swimming (2.4 miles), cycling (112 miles), and running (26.2 miles). The term “iron man” was taken from the “Hawaiian Ironman Triathlon” in 1978. The triathlon is recognized as an extremely high-intensity sport with physiological impact [1]. Triathletes are particularly prone to muscular injury and fatigue [2]. Exercise-induced muscle damage (EIDM) is mostly due to exercise-related oxidative stress, which results in a functional decrease in the muscles [3]. Mechanical stress and inflammatory stress also contribute to EIMD [4]. The intensive endurance could induce reactive oxygen species (ROS) because of excessive aerobic respiration, ischemia–reperfusion, and leukocyte activation [5]. Muscular and renal injuries have been observed during intensive endurance exercise, with increased oxidative stress and inflammation [6]. Dehydration and carbohydrate depletion also contribute to fatigue, and gastrointestinal (GI) problems, hyperthermia, and hyponatremia result in performance decrease during endurance exercise [7]. The central fatigue related to the central nervous system (CNS) might be mitigated with euglycemia maintenance by energy balance [8]. Thus, oxidative stress, inflammation, and energy balance could be critical factors to regulate the central and peripheral fatigue during endurance exercise.

Probiotic supplementation has also been shown to ameliorate allergic, metabolic, inflammatory, gastrointestinal, and respiratory conditions. Athletes often suffer from diarrhea, nausea, abdominal cramps, and bloating symptoms, especially during prolonged and exhaustive exercise, and gastrointestinal health could be critical for the regulation of exercise adaptation. Probiotic supplementation has been shown to improve the frequency, severity, and duration of respiratory infection and gastrointestinal illness, likely through interaction between the gut microbiota and immune system [9]. The combination of two probiotic strains (*Bifidobacterium breve* BR03 and *Streptococcus thermophilus* FP4) also help with exercise recovery following a bout of strenuous exercise [10]. However, another study focused on immune responses among young sedentary participants following resistance exercise combined with probiotics, but the probiotics did not produce an upregulation in immune responses following resistance exercise [11]. Two probiotic strains, *L. rhamnosus* IMC 501 and *L. paracasei* IMC 502, have had beneficial effects on athletic oxidative stress improvement during 4 weeks of intense training [12]. In addition, *L. plantarum* TWK10 and *Bifidobacterium longum* subsp. *longum* OLP-01 administration was able to accelerate the physiological exercise adaptation and elevate performance, possibly via energy harvesting and homeostasis, in previous studies [13,14]. These cited studies show that the tested probiotics have various physiological effects.

Probiotics have demonstrated positive effects on several health outcomes in adult health studies, and the five categories—gut microbiota changes, immune system response, lipid profile and cardiovascular disease risk, gastrointestinal discomfort, and reproductive health—have been reported on for the probiotic effects on health maintenance possible through transient improvement in gut microbiota concentration of specific bacteria, not persistent changes in microbiota [15]. Probiotic supplementation might be not always beneficial to health maintenance, and the particular patients, such as neonatal stages, malignancies, leaky gut, diabetes mellitus, and post-organ-transplant convalescence, could exacerbate the infectious conditions and antibiotics resistance by debilitating the immunity and plasmid-mediated transfer mechanisms with probiotic supplementation [16]. Therefore, the impacts of athlete’s microbiota by probiotics supplementation needed to be further investigated, and there have also been limited studies to reveal the probiotic supplementation on microbiota succession in special athlete population.

Based on our previous study, *L. plantarum* PS128 was also elucidated for the alleviation of exercise-induced oxidative stress and inflammation, and increases the performance recovery from fatigue [17]. We were interested in how the *L. plantarum* PS128 affects physiological adaptation during exercise and performance via modulation of microbiota in the current study. The different functional activities could also be further understood and revealed by the microbiota analysis.

## 2. Materials and Methods

### 2.1. Lactic Acid Bacteria

*Lactobacillus plantarum* PS128, provided by Professor Ying Chieh Tsai at Yang Ming University (Taipei, Taiwan), was cultivated and produced by Synbio Tech Inc. (Kaohsiung, Taiwan). Each capsule included 400 mg of lyophilized bacteria powder containing 300 mg of lyophilized *L. plantarum* PS128 powder (1.5 × 10^10^ colony-forming units (CFU)) and 100 mg microcrystalline cellulose as an excipient. The placebo group (PG) received the same type of capsules filled with 400 mg microcrystalline cellulose. The *L. plantarum* PS128 supplement group (LG) involved participants taking a single capsule twice per day, equivalent to 3 × 10^10^ CFU/day dosage.

### 2.2. Participants

In the current study, we recruited 20 participants with at least 5 years organized training from triathlon teams at Taipei City University, and randomly divided them into two groups: a placebo group (PG; *n* = 10) and an *L. plantarum* group (LG; *n* = 10), as a parallel-group study. The participants were randomly assigned into groups, and also required not to supplement the fermented food, probiotics, prebiotics, and antibiotics during the whole experimental process, and maintain a regular lifestyle, avoiding any strenuous exercise, staying up late, smoking, or consuming alcoholic beverages. All participants were male, and there were no significant differences in basal physical characteristics between the groups: age (21.6 ± 1.3 vs. 21.9 ± 1.4 years), weight (66.4 ± 7.7 vs. 66.0 ± 7.0 kg), height (167.2 ± 7.8 vs. 168.8 ± 7.3 cm), and maximal oxygen consumption (VO_2_max; 55.5 ± 8.6 vs. 56.6 ± 9.0 mL/kg/min) for the LG and PG groups, respectively. In the medical records, there were no fractures, dislocations, or lacerations 3 months before and during the experiment. The participants were also asked to provide written informed consent before participating in the study. The study was reviewed and approved by the Institutional Review Board of Taipei City University (Taipei, Taiwan; No. IRB-2015-025).

### 2.3. Experimental Design

This study was based on a double-blind experimental design to realize the possible effects of *L. plantarum* PS128 supplementation, and the study was designed as a parallel-group study to compare the probiotic supplement (LG) and placebo treatment (PG) after a 4-week intervention. The supplementation was combined with daily training for 4 weeks, and supplementation was scheduled for after training and before sleeping. The dietary records were analyzed for individual nutritional compositions and calories for reference. The feces, blood, and DEXA (dual-energy X-ray absorptiometer; GE Lunar, Madison, WI, United States) were collected only at the end of study for complete blood count (CBC), biochemistry, metagenome, related metabolites, and body composition after 4 weeks of supplementation. The endurance and VO_2_max were measured as part of the functional assessment.

### 2.4. VO_2_ Maximum and Endurance

Maximal oxygen consumption and exercise performance were evaluated with a treadmill (Pulsar, h/p/cosmos, Germany) and an auto respiratory analyzer K4b2 (Cosmed, Concord, CA, USA). Heart rate (HR) was also monitored using a Polar heart rate device. The speed and grade of the treadmill were simultaneously increased every 3 min until volitional fatigue, in accordance with the Bruce protocol [18]. Oxygen consumption was considered maximum when the respiratory exchange ratio (the volume ratio of carbon dioxide produced to oxygen consumed, VCO_2_/VO_2_) was above 1.10, and the maximum heart rate was achieved (maximum heart rate = 210 − age). The three largest VO_2_max values were averaged to obtain the VO_2_max of each volunteer.

### 2.5. Metagenome

The feces were immediately stored at −80 °C for bacterial DNA extraction. Feces were homogenized (MagNA Lyser System; Roche, Indianapolis, IN, USA) in ASL buffer, and total DNA was extracted directly using the QIAamp DNA Stool Mini Kit (Qiagen, Hilden, Germany), according to the manufacturer’s instructions. The hypervariable V1–V3 region of the bacterial 16S rRNA gene was amplified through polymerase chain reaction (PCR), with barcoded universal primers E8F (forward primer; 5′-AGAGTTT GATCMTGGCTCAG-3′) and 530R (reverse primer; 5′-CCGCGGCKGCTGGCAC-3′), and then finally added to the 454 Roche linker for sequencing. The PCR reaction condition was performed at 95 °C for 2 min, followed by 28 cycles at 95 °C for 30 s, 52 °C for 60 s, and 72 °C for 1 min, then held at 72 °C for 10 min, and finally maintained at 4 °C until use. PCR amplicons were purified using the NucleoFast 96 PCR Clean-Up Kit (Macherey–Nagel, San Diego, CA, USA), and quantified amplicons were directly sequenced using a Roche 454 GS FLX (LabX, Ontario, ON, Canada). The raw data obtained after the sequence was cleaned by pairing the raw tags and filtration, and then the chimera was removed to obtain the effective tags suitable for subsequent analysis. Then, operational taxonomic units (OTUs) with similarity greater than 97% were clustered and a species classification analysis performed. The sequences were further analyzed using a Ribosomal Database Project Naive Bayesian rRNA Classifier (version 2.5) to categorize the microbiota and investigate the relative proportions of microbiota existing in an indicated sample [19].

### 2.6. Body Composition

A dual-energy X-ray absorptiometer (DEXA) was applied to measure the body with a non-invasive test instrument. These procedures have been described in detail elsewhere [17].

### 2.7. Blood Sampling and CBC Analysis

Ten milliliters of venous blood were individually collected into collection tubes containing anticoagulant EDTA and Heparin. The plasma and serum without anticoagulant were centrifuged at 3000× *g* for 10 min. The plasma and serum were stored at −70 °C for further analysis. In addition, the complete blood count (CBC) profiles were also analyzed by KX-21 (Sysmex, Hyogo, Japan) for WBC, RBC, hemoglobin, hematocrit, platelet, and different lymphocytes.

### 2.8. Biochemical Variables

Glucose, total protein, albumin, aspartate aminotransferase (AST), alanine aminotransferase (ALT), lactate dehydrogenase (LDH), alkaline phosphatase (Alk-P), total cholesterol, triglyceride, blood urea nitrogen (BUN), creatinine, uric acid, high-density lipoprotein (HDL), and low-density lipoprotein (LDL) in the serum were quantified using an automatic biochemical analyzer (SIEMENS, Erlangen, Germany). Free fatty acids (BioVision, K612-100, San Francisco, CA, United States) in the serum were quantified using a colorimetric kit and enzyme-linked immunosorbent assay (ELISA) methods.

### 2.9. Short-Chain Fatty Acid (SCFA) Analysis

Fresh fecal samples were obtained from both groups only after 4 weeks of intervention. The fecal samples were weighed and suspended in 1 mL of water with 0.5% phosphoric acid per 0.1 g of sample, then homogenized for 2 min. The samples are then centrifuged (14.8 RPM for 10 min) and the supernatant collected. Next, an aliquot of ethyl acetate (300 μL) was added into the supernatant and mixed well before centrifuging (14.8 RPM for 10 min). The organic phase was collected for GC-MS analysis with an Agilent 5977B coupled with a 7693A autoinjector (Agilent Technologies, Palo Alto, CA, United States). The Nukol Capillary GC Column (30 m × 0.25 mm id, 0.25 μm df) was applied to the GC, and the gas (helium) carrier injected at 1 mL/min. The column temperature stared at 90 °C, then increased to 150 °C at 15 °C/min, to 170 °C at 5 °C/min, and finally to 250 °C at 20 °C/min for 2 min (total time, 14 min). The detector is operated in electron impact ionization mode (electron energy 70 eV), scanning the 30–250 *m*/*z* range. The indicated SCFA identification was based on the retention time of standard compounds and the assistance of the NIST 08 and Wiley7N libraries.

### 2.10. Statistics Comparison of Probiotic (Treatment) and Control Groups

All of the results were statistically analyzed using unpaired *t*-tests or Mann–Whitney U tests with SPSS 18.0 (IBM, New York, NY, United States). The values were expressed as mean ± SD, and the final numbers were rounded to the appropriate numerical presentation; *p*-values < 0.05 were considered significant.

## 3. Results

### 3.1. The Effects of PS128 on Exercise Performance and Body Composition

**Figure 1:**
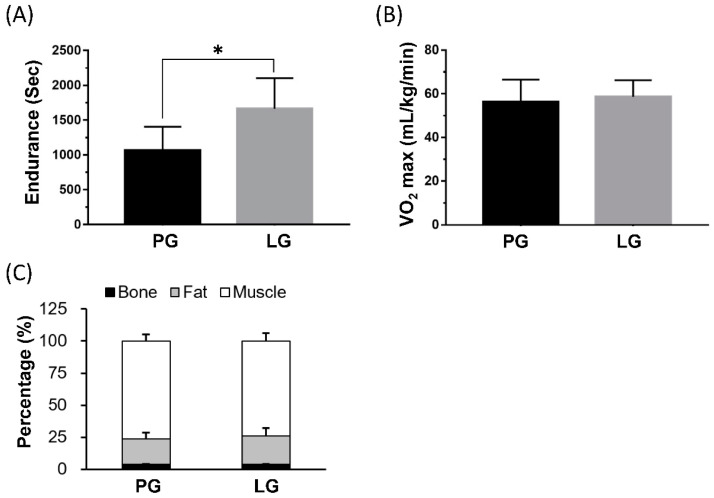
The effects of *L. plantarum* PS128 on exercise performance and body composition. The data are represented as mean ± SD. The endurance was record as exhaustive time with treadmill exercise, and maximal oxygen utilization (VO_2_max) was collected with gas exchange. PG and LG refer to the placebo and *L. plantarum* treatment groups, respectively. *: the significant difference between groups (*p* < 0.05).

The PS128 supplementation group (LG) could significantly elevate endurance performance by the treadmill exercise protocol; the LG’s performance could increase about 130% as compared to the PG group (*p* = 0.0035) (Figure 1A). However, at the end of the study, the VO_2_max and body composition (bone, fat, and lean percentage) demonstrated no significant difference between groups in the gas and DEXA analysis (Figure 1B,C).

### 3.2. The Effects of PS128 on Biochemical Variables and Complete Blood Count

The sampled blood was separated for complete blood count and biochemical variable analysis. There was no significant difference between groups in all indexes and biochemical variables (Table 1). In addiiton, the CBC was also not significantly different in white blood cells (WBCs), red blood cells (RBCs), subtype cells, hemoglobin (Hb), hematocrit (Hct), mean corpuscular hemoglobin (MCH), mean corpuscular hemoglobin concentration (MCHC), mean cell volume (MCV), and neutrophil-to-lymphocyte ratio (NLR), but the platelet concentration was significantly lower in the PG in the LG (Table 2).

### 3.3. Changes of the Relative Abundance at the Phylum and Genus Level

**Figure 2:**
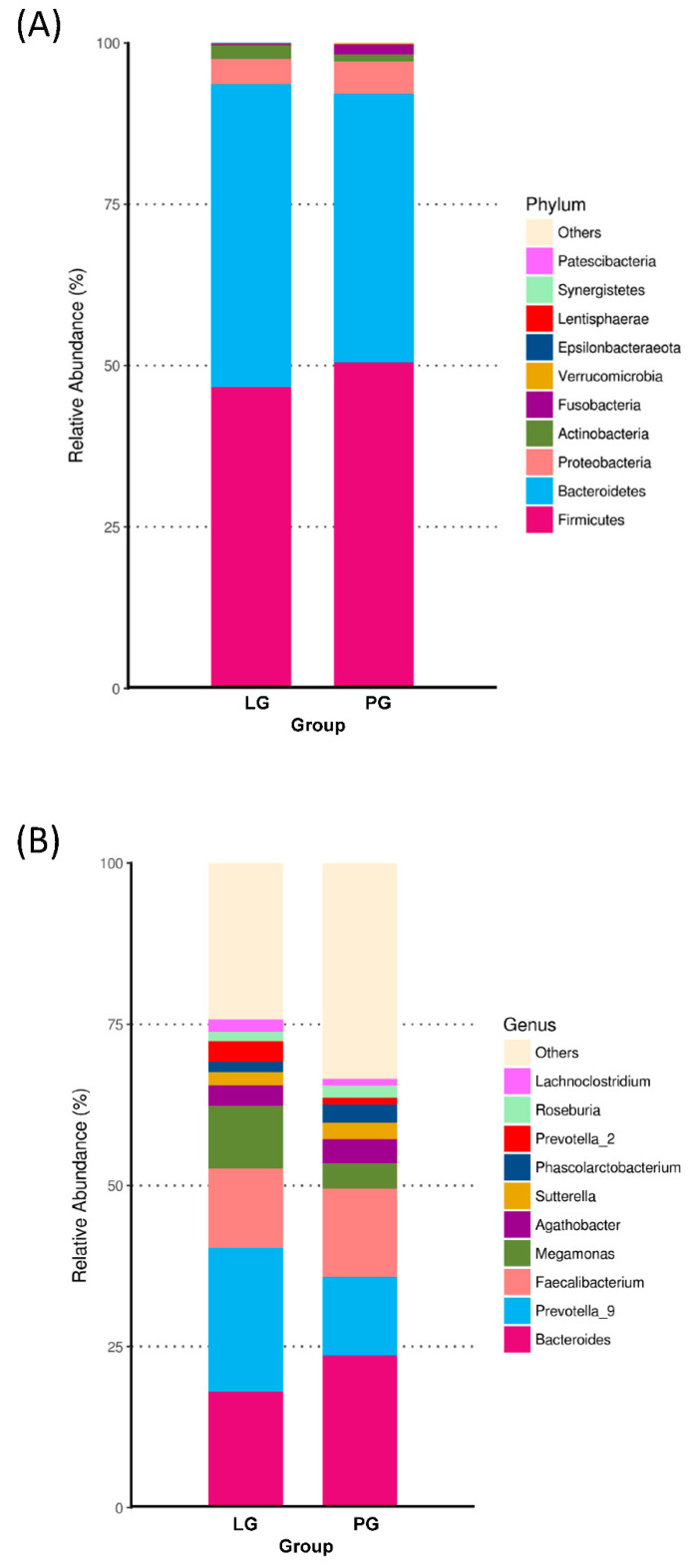
Changes of the relative abundance at the phylum and genus levels. Top 10 abundance at the phylum (**A**) and genus (**B**) level.

**Figure 3:**
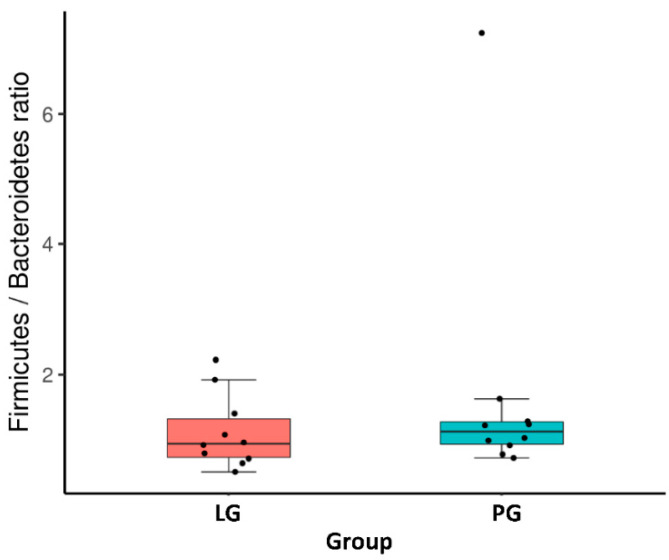
*Firmicutes*/*Bacteroidetes* ratio between groups.

At the phylum level, Figure 2A shows the ten most abundant bacteria for the LG and PG groups. With the different phyla occupying different dominant positions across two groups, Firmicutes and Bacteroidotes were the dominant phyla in the PG and LG. Even though there is no significant difference between the groups at the phylum level, the Firmicutes, Proteobacteria, Fusobacteria, Verrucomicrobia, Lentisphaerae, and Synergistetes represented a higher ratio in the PG (Figure 2A). The relative abundance of Bacteroidetes, Actinobacteria, and Epsilonbacteraeota increased in the LG as compared to the PG. In addition, the Firmicutes/Bacteroidetes ratio was not significantly different when comparing the LG and PG (Figure 3).

The relative abundances at the genus level are presented in Figure 2B. In the PG, *Bacteroides* was the most abundant genus, followed by the *Faecalibacterium*, *Prevotella*_9, *Megamonas*, *Agathobacter*, *Sutterella*, *Phascolarctobacterium*, *Lachnoclostridium*, and *Roseburia*. In the LG, the most abundant genera were *Prevotella*_9, followed by the *Bacteroides*, *Faecalibacterium*, *Megamonas*, *Agathobacter*, *Sutterella*, *Prevotella*_2, *Lachnoclostridium*, *Phascolarctobacterium*, and *Roseburia*.

### 3.4. The Significant Difference of Microbiota between Groups at the Genus Level

**Figure 4:**
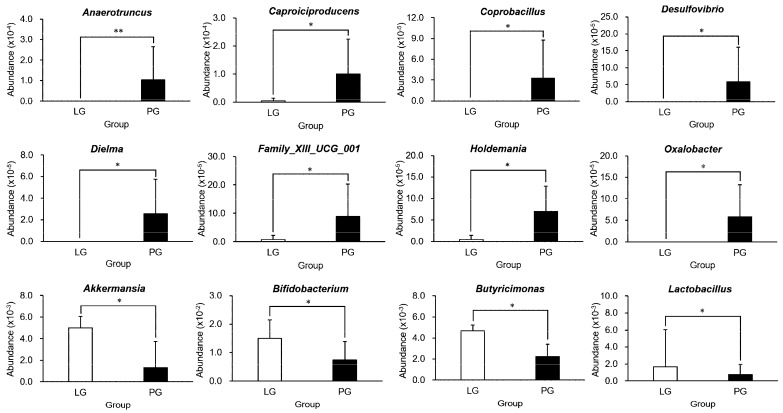
Relative abundance of predominant taxa at the genus level. Each value is presented as the mean ± SD, and the significance tests were conducted in each treatment population at the genus level. *: *p* < 0.05; **: *p* < 0.01.

In Figure 4, PS128 supplementation resulted in a significant difference in the abundance of predominant taxa at the genus level. The *Anaerotruncus*, *Caproiciproducens*, *Coprobacillus*, *Desulfovibrio*, *Dielma*, *Family_XIII_UCG_001*, *Holdemania*, and *Oxalobacter* genera were significantly decreased in the LG compared to the PG. Also, there was a significantly higher abundance of *Akkermansia*, *Bifidobacterium*, *Butyricimonas*, and *Lactobacillus* in the LG than in the PG.

### 3.5. Changes of Fecal Microbial Diversity

**Figure 5:**
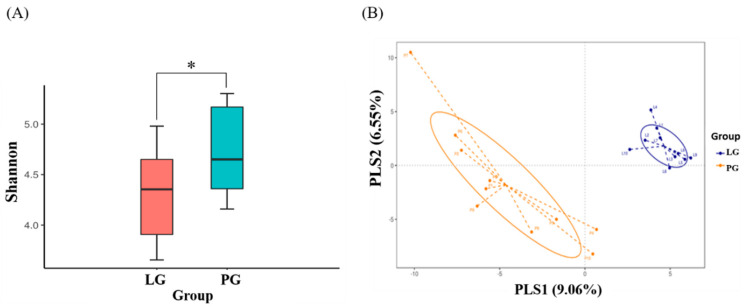
Changes of fecal microbial diversity: (**A**) Alpha diversity and (**B**) β diversity of athlete with *L. plantarum* PS128 (LG) and placebo (PG) treatment. *: the significant difference between groups; *p* < 0.05.

To assess fecal microbial community diversity, the Shannon index and partial least squares discriminant analysis (PLS-DA) were calculated. In Figure 5A, the Shannon index indicates that SP128 treatment (LG) significantly decreased the α-diversity of the intestinal microbial community as compared to the PG (*p* < 0.05). We also evaluated the compositions of the gut microbiota between the LG and PG using the score plots of the partial least squares discriminant analysis (PLS-DA) method. The plots showed that there was a significant separation between each group, with 9.1% and 6.6% explanation of the variation of the gut microbiota composition in PLS1 and PLS2, respectively (Figure 5B).

### 3.6. The Effects of PS128 on Short-Chain Fatty Acids

**Figure 6:**
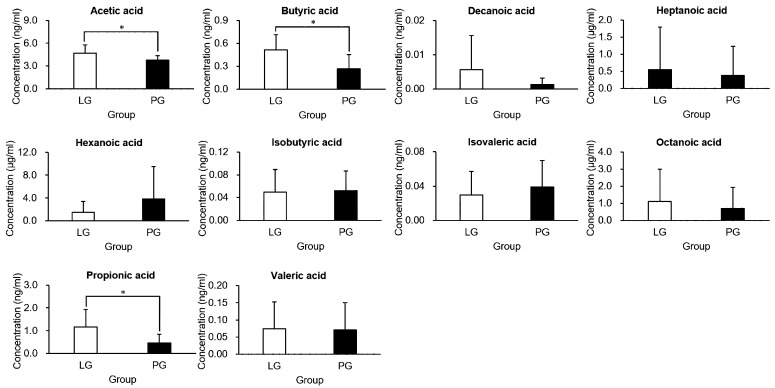
Short-chain fatty acid (SCFA) analysis in the feces of the triathletes. The LG and PG represented the triathletes receiving *L. plantarum* PS128 and placebo treatment groups, respectively, and *p*-values < 0.05 were considered significant (*) in each treatment population.

To further investigate the microbiota of both groups, feces were collected, and their short-chain fatty acid (SCFA) contents analyzed (Figure 6). Acetic, propionic, and butyric acid were the major SCFAs found in the feces. Compared with the PG, the LG had extremely elevated acetic acid, propionic acid, and butyric acid (*p* < 0.05). The other SCFAs (decanoic acid, heptanoic acid, hexanoic acid, isobutyric acid, isovaleric acid, octanoic acid, and valeric acid) were not significantly different when comparing groups.

## 4. Discussion

Athletes often attend off-site training and competitions. The environment, diet, and stress can cause gastrointestinal discomfort, including fistula, gastroenteritis, diarrhea, and constipation, and the maintenance of gut health is an important issue in preventive medicine [20]. In animal and human studies, the gut microbiota has been shown to play important roles in many aspects of health, including immune, metabolic, and neurobehavioral traits [21]. The microbiota of the gastrointestinal tract (GI) can also be considered as a virtual endocrine organ, producing its own panel of metabolites and including a mutual communication network between the gut and the brain [22]. The current study indicates that long-time supplementation with *Lactobacillus plantarum* PS128 resulted in a decrease of microbiota diversity, and an associated important microbiota change in the triathlete population. Therefore, probiotic supplementation could be an important nutrient strategy for promoting exercise performance in triathletes. In addition, the future study could be important to understand the effects of long-term probiotic supplementation for maintaining physiological homeostasis and GI health during endurance exercise, through functional microbiota modulation.

In the morbidity statistics of triathlons, gastrointestinal issues were the most common presentation (27.6%), followed by musculoskeletal injury (25.4%) and nonspecific dizziness (20.4%) [23]. GI distress might cause nutritional deficiency and physiological imbalance, and eventually lead to decreased performance, training efficacy, or drop-off of competition. Thus, a nutritional strategy could reduce GI severity, and the gut is an important organ for endurance athletes [24]. Probiotic supplementation could be considered as a nutritional application to ameliorate the GI syndrome by modifying the composition or activity of the microbiota, and also shows positive outcomes for several GI-related conditions [25]. In addition, *L. rhamnosus* GG supplementation could improve intestinal permeability by decreasing the inflammation cytokines in a sepsis-induced animal model [26]. *L. plantarum* PS128 also showed anti-inflammation activity after high-intensity endurance exercise [17], and it could be proposed to maintain the intestinal permeability for GI syndrome alleviation.

In a previous study, it was reported that prolonged physiological stress, such as military training, could increase the intestinal permeability related to microbiota change and inflammation [27]. A systematic review has also reported that exercise could cause the intestinal permeability elevation strongly correlated with the magnitude of exercise-induced hyperthermia [28]. The triathletes supplemented with *L. plantarum* PS128 had improved inflammation indexes [17]. In the current study, we observed that partial microbiota could be modulated by *L. plantarum* PS128, not only for the whole microbiota reconstruction, but also for its functional activities. In an animal study, *L. plantarum* PS128 could also modulate cerebral dopamine (DA) and serotonin (5-HT) to mitigate anxiety and depression behaviors [29]. The *L. plantarum* ZLP001 was also shown to increase the intestinal barrier function and exert anti-inflammation activities by means of strengthening the epithelium and modulating gut microbiota [30]. The lipoteichoic acid metabolite produced by *L. plantarum* K8 contributed to inflammation homeostasis through the modulation of Th1- and Th2-induced cytokines [31]. Therefore, in the current study, the microbiota was significantly affected by SP128, and could mitigate systematic inflammation and central fatigue associated with prolonged endurance performance.

In energy metabolism, the short-chain fatty acids (SCFAs), including acetate, butyrate, and propionate, are fermented and produced by microbiota from indigestible foods; they are not only the main energy source of colonocytes, making them crucial to gastrointestinal health, but also the main source of energy conversion and application [32]. In the current study, we found that SP128 supplementation could significantly elevate the plasma acetic, propionic, and butyric acid concentration and improve endurance performance. Okamoto et al. have shown that the microbiome could contribute to endurance exercise by producing SCFAs, especially acetate [33]. In other studies, acetate supplementation also resulted in an enhanced re-synthesis rate of muscle glycogen during the initial 4 h of the recovery period when compared with the control treatment [34]. Hernández et al. also addressed the biological functions of acetate, and reported that acetate from oral administration, acetogenic fiber, or probiotic supplementations beneficially affects host energy and metabolism relevant to insulin sensitivity, anti-inflammation, and glucose homeostasis via secretion of gut hormones, such as GLP-1 (glucagon-like peptide-1), peptide YY, and AMP activated protein kinase (AMPK) activation [35].

Microbiota compositions/portfolios are dynamic and can be affected by many factors, such as gender, age, lifestyle, behaviors, nutrition, medication, and infection [36]. Probiotics and prebiotics have been widely investigated and validated for their biological efficacy and health maintenance, but their mechanisms of actions require further study [37]. Exercise intervention could increase the diversity and *Bacteroidetes*/*Firmicutes* ratio of microbiota during diet-induced obesity, and athletes have also demonstrated a higher microbiota diversity than normal and obese populations [38,39]. A higher diversity of microbiota is generally considered to have beneficial effects on health status. In the current study, the microbiota diversity was relatively lower with supplementation with PS128 probiotics among triathletes, and the abundance of associated microbiota also contributed to the metabolite profile and endurance performance. According to the position of the International Society of Sports Nutrition, there is still much that is unknown about the effects of probiotics on the gut microbiota compositions of athletes [40]. The microbiota diversity was significantly lower in the LG than in the PG. However, the concept of microbial diversity was considered as an indicator of human health, but assumptions of increased diversity could be oversimplified for complicated interactive mechanisms of health and disease [41]. Thus, the athletes’ microbiota could be demonstrated for the different impacts with probiotic PS128 supplementation in the current study, and the specific microbiota abundance could be critical for the physiological functional activities. Compared to the PG, the LG had increased *Akkermansia*, *Bifidobacterium*, *Butyricimonas*, and *Lactobacillus*, and decreased *Anaerotruncus*, *Caproiciproducens*, *Coprobacillus*, *Desulfovibrio*, *Dielma*, *Family_XIII_UCG_001*, *Holdemania*, and *Oxalobacter* (Figure 4).

In previous studies, the colitis induced by TNBS (2,4,6-Trinitrobenzenesulfonic acid) or oxazolone model had revealed improvement in sulfate-reducing bacteria colonization, and *Desulfovibrio* could exacerbate the alterations with activation of the immune response and colitis development [42]. Also, high-fat diet-induced gut dysbiosis could modulate the abundance of *Oscillospira*, *Desulfovibrio*, *Coprobacillus*, and *Bilophila* associated with inflammation [43]. Another study has also demonstrated that oligosaccharides could ameliorate inflammation and its complications in high-fat diet-induced obesity by decreasing the abundances of the *Desulfovibrio*, *Anaerotruncus*, *Mucispirillum*, *Roseburia*, and *Odoribacter* [44]. Chronic kidney disease (CKD) could also be associated with systemic inflammation, due to the accumulation of ammonia from the urea metabolism pathway. Furthermore, it could further result in the decrease in the integrity of gut epithelial permeability and microbiota, such as *Holdemanella*, *Megamonas*, *Prevotella 2*, *Dielma*, and *Scardovia*, which are associated with the progression of CKD [45]. An upregulation of *Akkermansia* has been suggested to improve metabolic functions and help maintain gut permeability in disease-associated inflammation [46]. The SCFAs produced by *Bifidobacterium* spp. exert anti-inflammatory activities in mature enterocytes and immunocytes through the G-protein-coupled receptor and by inhibiting histone deacetylase 4 and 5 [47]. A previous study also demonstrated that stress and infection could result in a significant decrease in the SCFA-producing genera, such as *Butyricimonas*, *Anaerostipes*, *Butyricicoccus*, *Coprococcus*, and *Parabacteroides* [48]. *Akkermansia* and *Lactobacillus* showed a significant positive correlation with intestinal tight junction proteins and a negative correlation with inflammation and oxidative stress [49]. The triathletes participate in extremely high-intensity triathlon competition, and follow daily programmed training for higher sports-related physical fitness. During the training and competitive processes, immune suppression, oxidation, and inflammation, which are associated with the effects of microbiota and GI functions, represent a risk to the athlete’s performance and health maintenance. Taken together, these results suggest that modulation of the functional microbiota, through *L. plantarum* PS128, contributes to the regulation of inflammation, oxidation, and GI integrity, caused by high-intensity exercise, for physiological effects and athlete health, especially on the exercise adaptation.

The term VO_2_max refers to the maximum oxygen utilization during incremental exercise, and it usually represented aerobic capacity. In the current study, we detected a significant improvement in endurance capacity, but not in the VO_2_max index. These elite triathletes, having completed years of specialized training, have relatively high and stable VO_2_max values. According to previous studies, an effective training method, such as high-intensity interval training, can increase VO_2_max [50], and blood flow restriction during low-intensity rowing can also significantly improve the VO_2_max in elite rowers [51]. Regarding nutritional strategy (e.g., ketogenic diet and vitamin D supplementation), the VO_2_max was not significantly elevated in endurance athletes and military recruits [52,53]. Exercise training seems to be more effective than nutritional strategies for the improvement in VO_2_max index. The significant increase in endurance performance in the probiotics supplement group could also be considered as the amelioration of central and peripheral fatigue mechanisms. Probiotics, such as TWK10 and PS128, have roles in energy harvesting, the regulation of inflammation and oxidative stress, and cerebral hormones, which were associated with fatigue regulation for higher endurance performance [15,17,29]. Therefore, we believe probiotics have limited usefulness for increasing VO_2_max, but are effective for reducing oxidative stress and inflammation and contributing to energy maintenance for improved endurance capacities. In addition, in the current study we also observed that the probiotic supplementation did not affect the VO_2_max, but acted through the modulation of the athletes’ microbiota.

The probiotics still should be prudent for therapeutic application, because of the outcomes from the meta-analyses and randomized controlled trials. These are that specific strains or specific prebiotic compounds provide a specific health effect in a particular patient group from a particular population, while medical effects and generalizations about “probiotics” should be avoided [54]. In the current study, we observed the PS128 could exert functional activities on physiological adaptation and exercise performance through the potential microbiota changes in athlete population. However, similar effects should be further elucidated in other population, such as healthy adults, the elderly, and diseased populations. Also, the partial probiotics demonstrated detrimental effects by the mean of capacity for inducing local and systemic adverse effects, contradicting their definition as beneficial for human health. Therefore, more caution, safety exploration, and stringent regulation should be further evaluated to prevent these deleterious effects [55]. In the current study, the biochemistries, complete blood cell analysis, and basic physiological characteristics did not show a significant difference between groups, but the detailed pathological observations and immune responses should be further validated by animal experimental designs for health consideration.

In current probiotics, the genera *Bifidobacterium* and *Lactobacillus* have frequently been credited for health benefits. The probiotics applied to marathon runners could ameliorate GI syndromes and reduce the risk of upper respiratory illness [56]. However, most of these studies have not addressed much on how the individual microbiota changes and what are the concessions from probiotic supplementation among athletes. Probiotic supplementation could also modulate microbiota diversity and abundance. *L. plantarum* PS128 supplementation might also have functional activities and an anti-inflammation effect through modulation of the microbiota.

## 5. Conclusions

Based on our previous and current findings, we believe that *L. plantarum* PS128 supplementation—via modulation of microbiota—ameliorates oxidative stress and inflammation and improves performance under high-intensity exercise. Supplementation with *L. plantarum* PS128 reduced microbiota diversity and modulated the athletes’ microbiota, with both relative decreases (*Anaerotruncus*, *Caproiciproducens*, *Coprobacillus*, *Desulfovibrio*, *Dielma*, *Family_XIII*, *Holdemania*, and *Oxalobacter*) and increases (*Akkermansia*, *Bifidobacterium*, *Butyricimonas*, and *Lactobacillus*) in genera. *L. plantarum* PS128 could be potentially applied to triathletes to maintain physiological homeostasis and GI health during endurance exercise through functional microbiota modulation. However, more efficacy, mechanism, and safety studies with PS128 supplements should be further elucidated on different clinical populations and animal designs in the future.

## Figures and Tables

**Table 1:** Blood biochemistry analysis.

Parameters	PG	LG	*p*-Value
Albumin (g/dL)	4.7 ± 0.2	4.8 ± 0.2	0.691
Alk-P (U/L)	70.6 ± 21.3	76.7 ± 18.7	0.505
BUN (mg/dL)	16.5 ± 3.2	14.8 ± 2.7	0.072
Creatinine (mg/dL)	1.1 ± 0.1	1.01 ± 0.1	0.109
Free Fatty acid (ng/mL)	11.0 ± 2.8	11.7 ± 2.3	0.561
AST (U/L)	25.7 ± 14.5	21.8 ± 4.3	0.970
ALT (U/L)	13.9 ± 6.2	12.5 ± 2.8	0.970
HDL Cholesterol (mg/dL)	50.6 ± 4.7	50.7 ± 5.1	0.964
LDH (U/L)	268.8 ± 64.9	270.6 ± 50.5	0.946
LDL Cholesterol (mg/dL)	101.9 ± 17.8	107.5 ± 28.0	0.600
Serum AC Sugar (mg/dL)	82.1 ± 14.6	76.3 ± 20.2	0.471
T-Cho/HDL-Cho	3.3 ± 0.5	3.4 ± 0.6	0.744
Total Cholesterol (mg/dL)	166.9 ± 18.0	173.0 ± 34.8	0.629
Total Protein (g/dL)	7.2 ± 0.4	7.3 ± 0.5	0.582
Triglyceride (mg/dL)	111.9 ± 46.1	107.5 ± 50.1	0.840
Uric Acid (mg/dL)	5.8 ± 1.1	5.9 ± 1.0	0.833

LG and PG represent the *L. plantarum PS128* and placebo treatment groups, respectively. LDH: lactate dehydrogenase, Alk-P: alkaline phosphatase, BUN: blood urea nitrogen, AST: aspartate aminotransferase, ALT: alanine aminotransferase.

**Table 2:** Complete Blood cell analysis.

Parameters	PG	LG	*p*-Value
Hb (g/dL)	15.1 ± 1.6	14.8 ± 1.3	0.595
Hct (%)	15.0 ± 4.5	44.3 ± 3.9	0.571
MCH (pg)	30.4 ± 1.7	30.5 ± 1.2	0.988
MCHC (g/dL)	33.4 ± 0.4	33.4 ± 0.2	1.000
MCV (fL)	90.9 ± 4.4	92.1 ± 4.0	0.632
WBCs (10^3^/μL)	8.5 ± 2.0	8.5 ± 2.2	0.731
Basophil (%)	0.8 ± 0.2	0.9 ± 0.3	0.349
Eosinophil (%)	4.4 ± 2.7	3.4 ± 2.6	0.571
Lymphocyte (%)	21.0 ± 5.4	21.2 ± 4.8	0.982
Monocyte (%)	8.3 ± 2.1	8.9 ± 3.4	0.674
Neutrophil (%)	65.5 ± 7.9	65.6 ± 7.0	0.985
Platelet (10^3^/μL)	247.2 ± 32.7	289.6 ± 50.4	0.049
RBCs (10^6^/μL)	5.0 ± 0.5	4.9 ± 0.5	0.649
NLR	3.4 ± 1.4	3.3 ± 1.2	0.940

LG and PG represent the *L. plantarum* PS128 and placebo treatment groups, respectively. Hb: hemoglobin; Hct: hematocrit; MCH: mean corpuscular hemoglobin; MCHC: mean corpuscular hemoglobin concentration; MCV: mean cell volume; WBCs: white blood cells; RBCs: red blood cells; NLR: neutrophil-to-lymphocyte ratio; PLR: platelet-to-neutrophil ratio.
